# Epigenetic Factors in Pathogenesis of Retinoblastoma: DNA Methylation and Histone Acetylation

**DOI:** 10.3390/cimb47100844

**Published:** 2025-10-14

**Authors:** Georgios Kiosis, Kanellos Skourtsidis, Despoina Ioannou, Vasilis-Spyridon Tseriotis, Konstantinos Stergiou, Fani Akritidou, Theodora Papamitsou, Maria Kourti, Sofia Karachrysafi

**Affiliations:** 1 Research Team “Histologistas”, Interinstitutional Postgraduate Program “Health and Environmental Factors”, Medical School, Faculty of Health Sciences, Aristotle University of Thessaloniki, 541 24 Thessaloniki, Greece; gkiosis@auth.gr (G.K.); dioana@auth.gr (D.I.); konstantinos.d.stergiou@outlook.com (K.S.); fani-ak@hotmail.com (F.A.); thpapami@auth.gr (T.P.); sofia_karachrysafi@outlook.com (S.K.); 2 Medical School, Faculty of Health Sciences, Aristotle University of Thessaloniki, 541 24 Thessaloniki, Greece; 3 Department of Neurology, General Hospital of Thessaloniki “Agios Pavlos”, 551 34 Thessaloniki, Greece; vasilistseriotis@hotmail.com; 4 Laboratory of Clinical Pharmacology, Medical School, Aristotle University of Thessaloniki, 541 24 Thessaloniki, Greece; 5 Laboratory of Histology-Embryology, Medical School, Aristotle University of Thessaloniki, 541 24 Thessaloniki, Greece; 6 Pediatric & Adolescent Hematology Oncology Unit, 2nd Pediatric Department, Faculty of Health Sciences, Aristotle University of Thessaloniki, AHEPA Hospital, 546 36 Thessaloniki, Greece

**Keywords:** retinoblastoma, epigenetics, DNA methylation, histone acetylation

## Abstract

(Background) Retinoblastoma is the most common intraocular malignancy in childhood, primarily caused by mutations in the *RB1* gene. However, increasing evidence highlights the significant role of epigenetic mechanisms, particularly DNA methylation and histone acetylation, in tumor initiation and progression. This review aims to summarize and critically assess recent findings on how DNA methylation and histone acetylation contribute to the pathogenesis of retinoblastoma, and to explore their potential role as diagnostic biomarkers and therapeutic targets. (Methods) We searched the databases PubMed, Scopus, and ScienceDirect following PRISMA guidelines. Eligible studies were English-language, open-access articles published within the last ten years, including cohort studies, research articles, and case reports. After rigorous screening, 18 studies were included in the final analysis. (Results) Aberrant DNA methylation was found to inactivate tumor suppressor genes (*RB1, RASSF1A, p16INK4A, MGMT*) and promote oncogenesis through hypermethylation of regulatory elements. Similarly, histone acetylation’s dysregulation contributed to chromatin remodeling and overexpression of oncogenic factors such as SYK, GALNT8, and lincRNA-ROR. Elevated histone deacetylase (HDAC) activity was also linked to tumor cell proliferation, metastasis, and treatment resistance. Epigenetic inhibitors targeting these pathways demonstrated promising therapeutic potential. (Conclusions) DNA methylation and histone acetylation play a crucial role in the epigenetic regulation of genes implicated in retinoblastoma. Their dysregulation promotes tumorigenesis, and targeting these mechanisms represents a promising avenue for novel diagnostic and therapeutic strategies in pediatric oncology.

## 1. Introduction

Retinoblastoma is a malignant tumor of the retina, originating from the mature precursor of the cone in the developing retina [1]. It usually occurs in children before the age of five, with an incidence of approximately 1 in 15,000–20,000 live births [2]. Retinoblastoma shows no geographic, racial, or sex predilections and accounts for only 3–4% of all childhood cancers [2,3,4]. Nevertheless, it is the most common intraocular malignancy in pediatric patients, responsible for 6% of cancers in children and 5% of blindness in pediatric populations [1,2].

Retinoblastoma significantly impacts patients’ sight, appearance, and mental health, and in advanced stages, metastasis can be life-threatening [5]. The disease can be unilateral (60% of cases, mean age of diagnosis 24 months) or bilateral (40% of cases, mean age of diagnosis 15 months) [1,2]. Tumor spread often occurs along the optic nerve into the brain, facilitating metastasis [1,6,7]. Early diagnosis is crucial: a delay of more than six months from the first clinical signs increases mortality by about 70% [8], while timely detection improves both survival and vision outcomes [8].

Survival rates vary widely across the globe. In high-income countries, mortality is as low as 3–5%, with three-year survival approaching 99–100% [2,9,10]. In contrast, in low- and middle-income settings, mortality reaches 40–70%, and three-year survival stands at only 60%. This disparity is largely attributable to health system-related factors, including delayed diagnosis; limited access to specialized oncology and ophthalmology services; lack of infrastructure for chemotherapy, enucleation, and focal therapies; as well as socioeconomic barriers to treatment adherence. Current evidence suggests that biological or clinical differences in tumor behavior are not the primary drivers of this survival gap [2,9,10].

Current clinical management includes laser therapy, cryotherapy, radiotherapy, systemic chemotherapy, and enucleation. Additionally, intravitreal injections of agents such as melphalan and topotecan are employed, though these carry risks of adverse effects including vitreous hemorrhage, secondary uveitis, and secondary glaucoma [11,12].

Retinoblastoma can occur as either a heritable or sporadic disease. Heritable cases represent one third of all patients, presenting as unilateral or bilateral tumors and often associated with other malignancies [13]. Sporadic cases are typically unilateral [1,2]. Sporadic cases are not stratified by inheritance but by clinical and staging criteria—such as tumor size, location, seeding, and spread—to assess risk. Commonly used systems include the International Classification of Intraocular Retinoblastoma (IIRC/ICRB), the International Retinoblastoma Staging System (IRSS), and the American Joint Committee on Cancer Tumor Nodes Metastasis (AJCC TNMH) staging system—all of which apply uniformly to both heritable and sporadic retinoblastoma to guide prognosis and treatment decisions [14,15,16].

The disease is primarily driven by mutations in the tumor suppressor gene *Retinoblastoma 1 (RB1)*, located on chromosome 13q14, yet alterations in other genomic loci have also been reported as pathogenic [1,2]. A systematic search in ClinVar using the term ‘retinoblastoma’ and applying the pathogenic filter retrieved a total of 789 entries. As expected, the vast majority of these pathogenic variants were identified in *RB1*, reaffirming its central and indispensable role in retinoblastoma pathogenesis. Nevertheless, a smaller proportion of variants were mapped to additional genes and genomic loci, including *ITM2B*, *AKAP11*, *ALG11*, *LPAR6*, *MED4*, and several uncharacterized regions (*LOC124885096*, *LOC130009754*, *LOC130009755*). Although the functional relevance of these non-RB1 loci to retinoblastoma remains largely uncertain, their annotation in ClinVar highlights the possibility that broader genomic contexts may occasionally be implicated. Beyond genetic alterations, epigenetic modifications have also been implicated in retinoblastoma pathogenesis [1,2,17].

Epigenetics refers to heritable changes in gene expression that occur without alterations in the DNA sequence [1,2,18]. Increasing evidence highlights the contribution of epigenetic dysregulation to retinoblastoma tumor progression [19,20,21]. Epigenetic alterations of *RB1* affect not only the transcriptional levels of the gene but also the functional ability of protein of Retinoblastoma (pRb) to impose cell cycle control and cellular senescence, which are two processes that act as natural barriers to tumorigenesis. Through modulation of the RB–E2 promoter-binding factor (E2F) pathway, pRb prevents the G1/S transition and cooperates with other critical effectors such as p53 and p16^INK4a to establish an irreversible growth arrest characteristic of senescence. Epigenetic silencing of *RB1* disrupts these mechanisms, resulting in loss of senescence enforcement and enabling uncontrolled proliferation and malignant transformation [22].

Two major epigenetic mechanisms include DNA methylation and histone acetylation. DNA methylation, catalyzed by DNA methyltransferases (DNMTs), represses gene expression by adding methyl groups to cytosine bases. In retinoblastoma, hypermethylation of RB1 occurs in about 9% of unilateral sporadic tumors and can affect any of its 27 cytosine–phosphate–guanine (CpG) sites [21,23]. The promoter/exon 1-associated CpG island (*CpG106*) overlaps the transcription start site and exon 1 (GRCh38: chr13:48,877,485–48,879,028; GRCh37: chr13:48,509,467–48,511,010) [24]. Additionally, a 1.2 kb intron 2 CpG island (*CpG85*), which exhibits parent-of-origin-specific differential methylation, maps to (GRCh38: chr13:48,889,885–48,891,227; GRCh37: chr13:48,521,867–48,523,209) [25]. Additional genes are also subject to hyper- or hypomethylation, contributing to oncogenesis [2,18,26,27,28,29].

Histone acetylation is another key modification in retinoblastoma pathogenesis [1,2,30,31]. Histone acetyltransferases (HATs) and histone deacetylases (HDACs) regulate chromatin structure by adding or removing acetyl groups [31,32,33,34]. Acetylation relaxes chromatin (euchromatin), enhancing transcription, while deacetylation promotes chromatin condensation and transcriptional repression [31,35,36]. Dysregulation of this balance disrupts cell proliferation and death, facilitating retinoblastoma initiation and progression [1,31,32,35].

In this study, we aim to summarize and analyze recent data from the last decade on the role of DNA methylation and histone acetylation in the pathogenesis of retinoblastoma. Unlike previous reviews that primarily focused on genetic factors or provided fragmented insights into epigenetic mechanisms, our work concentrates specifically on these two key epigenetic processes. By highlighting their potential as diagnostic biomarkers and therapeutic targets, this review offers a focused and clinically relevant perspective that may support the development of future personalized therapies for retinoblastoma.

## 2. Materials and Methods

### 2.1. Search Strategy and Eligibility Criteria

We conducted a search in PubMed/MEDLINE, Scopus and ScienceDirect databases covering all the papers published from 2015 until 19 April 2025. The search was conducted using keywords “retinoblastoma”, “epigenetics”, “acetylation”, “methylation”, as well as synonymous terms.

### 2.2. Eligibility Criteria

We included studies that correlated histone acetylation or DNA methylation and the development of retinoblastoma. Eligible publication types included original research articles, cohort (retrospective, prospective) studies and case reports, open access, published in the last 10 years, and in the English language.

### 2.3. Data Management

**Figure 1:**
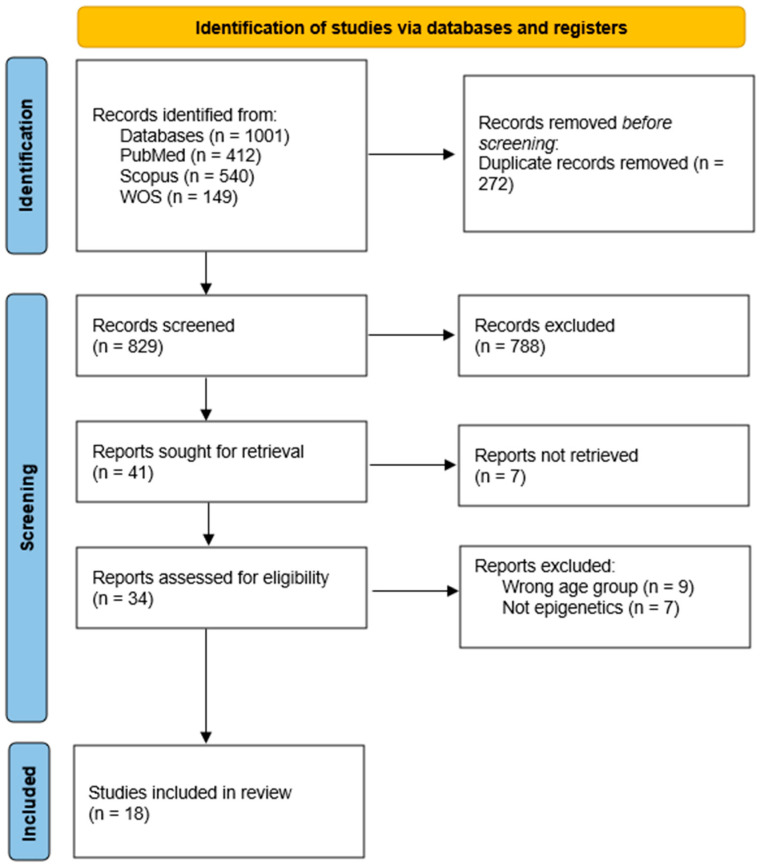
PRISMA Flow Diagram. From [37]. For more information, visit: http://www.prisma-statement.org/ (accessed on 19 June 2025).

Initially, the total number of articles found without applying the criteria was recorded. The total number of articles found was 1101, with 412 being from PubMed, 540 from Scopus, and 149 from Web of Science. A total of 272 articles were duplicates, so 829 articles were screened by titles and abstracts for eligibility. After the first screening was applied, 788 articles were excluded as ineligible. These 788 articles were excluded for one or more of the following reasons: lack of relevance to the topic (not addressing retinoblastoma or epigenetic regulation), absence of original data (e.g., editorials, letters, non-systematic reviews), language other than English, and restricted accessibility (non-open access). Thus, 41 articles were recorded and sought for retrieval. A total of 7 reports were not retrieved due to restricted access or unavailability of the full text. Moving on, 34 final articles were assessed for eligibility, from which a total of 15 articles were excluded for the following reasons—9 articles were excluded because the wrong age group was used (not referring to kids and adolescents), and 7 due to not being relevant to epigenetics. Finally, 18 articles were included in our review. The PRISMA flow diagram was constructed and presents the progress from our initial studies to final results (Figure 1).

## 3. Results

### 3.1. Methylation

#### 3.1.1. Tumor Suppressive Molecules Affected

Multiple tumor suppressor genes are silenced via hypermethylation in retinoblastoma, leading to uncontrolled proliferation through disruption of cell cycle control, apoptosis, and DNA repair mechanisms. According to Jie Sun et al. 2020, [6] methylation of CpG islands in the *RB1* gene is associated with retinoblastoma. These regions are part of the promoter of the gene or belong to its first exon [38]. Hypermethylation of these regions leads to decreased expression of the tumor suppressor gene *RB1* [39]. Moreover, this study claims that beyond *RB1*, methylation of the tumor suppressor gene *RASSF1A* which is related to the stability of the microtubules [40], as well as p16INK4A which encodes a CDK inhibitor that stops the cell cycle [41], can lead to retinoblastoma. Also, another important enzyme that is associated with this eye cancer is MGMT (O6- methylguanine-DNA methyltransferase), a DNA repair enzyme. Hypermethylation of its promoter can lead to bilateral retinoblastoma because the sensitivity of glioma to alkylating agents is increased. Another gene whose promoter’s hypermethylation is a sign of retinoblastoma is the *Trefoil Factor Family gene (TFF1),* a gene that encodes a protein that regulates the epithelial integrity of the retina cells. Downregulation of *TFF1* due to promoter hypermethylation contributes to tumor development by impairing apoptotic mechanisms involving p53 [42].

The study by A. M. Raizis et al. 2021, [21] also states that hypermethylation of CpG islands in the promoter of *RB1* is associated with retinoblastoma, which is in agreement with Jie Sun et al. 2020 [6]. But they also found out that hypermethylation in these regions is less common in heterozygous *RB1* promoter variants. Also, they state that CpGs upstream of the *RB1* core promoter are hypomethylated, probably because of the reduced HDAC [21,43].

The Özge Şükrüoğlu Erdoğan et al. 2024 [23] study comes in contrast to the other studies that are included in this review. This study mentions that there is no significant difference in the methylation of *RB1* promoter between retinoblastoma patients and healthy control groups. Also, this study revealed that the methylation of *RB1* promoter is not associated with hereditary retinoblastoma. The discrepancies between the findings of Sun et al. [6] and Raizis et al. [21] compared to Erdoğan et al. [23] may be attributed to differences in study design, patient cohorts, and methodological approaches. It is necessary to mention that the authors of this study support that their results are exported from a small sample because of the rarity of retinoblastoma and that all participants in their study did not possess germline *RB1* gene mutations but had a familial background of retinoblastoma. Furthermore, this inconsistency between articles may reflect the heterogeneity of retinoblastoma subtypes. Thus, while promoter hypermethylation appears to play a role in certain subsets of patients, its contribution may not be universal across all clinical presentations of retinoblastoma.

The Hülya Yazici et al. 2020 [26] study also mentions the connection between higher global genome methylation levels and the presence of retinoblastoma. To be more certain, these levels were significantly augmented (1.5-fold higher) in the peripheral blood and cancer tissues of retinoblastoma patients. The absence of retinoblastoma protein (pRb) and retinoblastoma associated protein E2F leads to the overexpression of DNA methyltransferase 1 (DNMT1) [44,45]. Overexpression of DNMT1 is associated with hypermethylation of tumor suppressor genes as well as inactivation of other genes and genomic regions that may lead to loss of heterozygosity (LOH) in the other RB1 allele in cases of retinoblastoma when only one of the two alleles was not functional [46].

The Lan Jin et al. 2021 [27] study states that the expression of *Paired Box Gene 5 (Pax5)* was decreased in retinoblastoma patients. This was a result of the higher methylation level of the *Pax5* gene in retinoblastoma cells, rather than normal retinal epithelial cells. The results of this study showed that the methylation of *Pax5* was inhibited by cyclophosphamide (CTX) and downregulated the expression of DNMTs. The upregulated *Pax5* further inhibited the proliferation, migration, and invasion of retinoblastoma cells, and promoted cell apoptosis. It is known that *Pax5* inhibits the growth of retinoblastoma by inhibiting the activity of the Notch1 signaling pathway [47].

The Bo Yang et al. 2020 [28] study states that decreased expression of miR34a due to its hypermethylation is present in retinoblastoma cells. This increased methylation was promoted due to *Cancer Susceptibility Candidate 8 (CASC8)* overexpression. It is stated that overexpression of *CASC8* as well as decreased production of miR34a are associated with cell proliferation and thus with retinoblastoma. miR34a targets multiple oncogenic genes, such as *Cluster of Differentiation 44 (CD44)* and *Mesenchymal–Epithelial Transition factor (c-MET)*, by suppressing cell proliferation, metastasis, and cancer growth [48,49].

#### 3.1.2. Oncogenic Molecules Affected

In addition to the silencing of tumor suppressor genes through hypermethylation, several studies have shown that aberrant methylation can also lead to the activation of oncogenic pathways. In such cases, methylation does not suppress gene expression but instead contributes to the overexpression of oncogenes that promote uncontrolled proliferation, metastatic potential, and resistance to apoptosis. The bioinformatic analysis by Yuyang Zeng et al. 2020 [18] discusses two genes which are hypermethylated in retinoblastoma and function as oncogenes. The first is *Kinesin Family Member 14 (KIF14)* which encodes kinesin family member 14, a motor protein involved in critical cellular processes, especially during mitosis and cytokinesis. It plays an essential role in ensuring proper chromosome segregation and cell division. Although DNA methylation typically suppresses gene expression, *KIF14* is differentially methylated and overexpressed in retinoblastoma, suggesting complex epigenetic regulation. Hyperexpression of this protein is associated with other types of cancer too [50,51,52]. The second gene is *MCM6*, which encodes a protein that is a part of the minichromosome maintenance (MCM) protein complex. In retinoblastoma, hypermethylation of *MCM6* leads to reduced expression, which can disrupt normal DNA replication control and contribute to tumorigenesis by affecting cell cycle regulation [18]. This mechanism is supported by studies in other cancers showing that methylation-dependent repression of *MCM6* can influence cell proliferation and metastasis [53].

The Sipeng Zuo et al. 2023 [17] study reveals a connection between N5-methylcytosine (m5C), a post-transcriptional RNA modification, and retinoblastoma. This methylation pattern of tRNAs, rRNAs and mRNAs is catalyzed by enzymes in the NOL1/NOP2/SUN domain (NSUN) family and DNA methyltransferase DNMT2 [54]. Enhanced NSUN2 was related with elevated levels of m5C modification and, accordingly, with excess cell proliferation and invasion [55]. All the retinoblastoma samples showed increased global m5C levels in contrast to retina samples from non-retinoblastoma patients. This overexpression affects the biosynthesis of purines by enhancing their production. The mechanism that leads to significantly enhanced nucleotides production is associated with phosphoribosylformylglycinamidine synthase (PFAS), a target of NSUN2. PFAS fuels purine synthesis in retinoblastoma cells by supporting the content of several intermediate metabolites, including IMP, AMP and GMP [56]. NSUN2 modulates the PFAS m5C modification level and potentially enhances PFAS expression. Hypermethylation of m5C in PFAS mRNA turns them into oncogenes that are highly expressed in retinoblastoma [57].

According to the Peiyao Mao et al. 2022 [23] study, there are two subtypes of retinoblastoma patients, high immune cell infiltration (ICI) and low ICI. Hypermethylation is present in both subtypes, but some differences exist. In the high-ICI group most hypermethylated probes were located in the open sea, followed by N-shore and S-shore regions. In the low-ICI subgroup, the majority of hypermethylated probes were located in the open sea too, but they were followed by CpG islands and N-shore regions. The study also mentions the connection between *BIRC5 (survinin)* and DNA methylation in retinoblastoma regulated by Dnmt1, Dnmt3a, and Dnmt3b. Moreover, in the high-ICI subgroup, *CD83, HLA-DOA, IRF4, DOK3,* and *CXCR1* genes were also hypermethylated and thus associated with the presence of retinoblastoma [58].

The Qi Zeng et al. 2021 [29] study reveals two more CpG loci that are hypermethylated in retinoblastoma. Both of these CpG loci are associated with a transcription factor named TFAP2A. Also, TFAP2A and cfDNA were found to be hypermethylated and the study states that the methylation levels of these two molecules could be diagnostic biomarkers of retinoblastoma. TFAP2A is a transcription factor that is expressed in neural c cell lineage and is associated with the neural tube closure and the development of facial characteristics [59,60].

The Hiroshi Tanaka et al. 2020 [61] study found that Nuclear Receptor-Binding SET Domain Protein 2 (NSD2) could methylate Aurora Kinase A (AURKA). Methylated AURKA interacts with p53 and induces proteasome-mediated degradation of p53. On the contrary, loss of methylation of AURKA results in p21 induction and thus apoptosis of cancer cells and limited cell proliferation [62,63].

The Tatsiana Ryl et al. 2024 [7] study analyzed the methylation profiles in two retinoblastoma subtypes with differences in clinical characteristics, genomic alterations, and prognoses. Subtype 1 retinoblastoma is characterized by fewer gene alterations than subtype 2. The surrogate marker for subtype 2 is overexpression of *TFF1*. They also categorized retinoblastoma patients in three clusters labelled A, B and C. Hypermethylation was present in probes of type B retinoblastoma, while hypomethylation was found in cluster C. The methylated regions were also CpG regions in all cases. Hypermethylated genes in cluster B were involved in camera-eye development, neuronal function, and synapse organization. Cluster C was enriched with hypomethylated genes involved in smell perception. The E-box (bHLH-binding motifs) variants, CACCTG and CATCTG, were enriched in minimal regions around hypomethylated CpGs in cluster C, indicating a potentially more open chromatin configuration for v-myc avian myelocytomatosis viral oncogene neuroblastoma-derived homolog (MYCN)-driven gene regulation [64]. Moreover, the bHLH transcription factor NHLH1 was hypomethylated and thus overexpressed in cluster C [7].

The most important findings regarding DNA methylation are presented in Table 1.

### 3.2. Acetylation

#### 3.2.1. Histone Acetyltransferase (HAT)-Mediated Activation

While histone acetylation is generally associated with transcriptional activation and open chromatin, in retinoblastoma this process can become pathologically redirected toward oncogene amplification. According to Linbin Zhou et al. 2024 [2] two acetylation marks, H3K9ac and H3K27ac, at the *GAU1/GALNT8* site in retinoblastoma cells lead to oncogenesis due to the open chromatin conformation at this site, which was caused by acetylation [65]. Overexpression of the oncogene *Spleen Tyrosine Kinase (SYK)*, was associated with acetylation of histone H3K9/14ac, located at the promoter of this gene, in retinoblastoma cases [2]. Finally, as Xiangyi Ma et al. 2024 [1] also reports, in retinoblastoma cells, there was increased expression of IincRNA-ROR. This is the result of acetylation of histone H3K27 by the HAT binding protein CREB at the promoter of IincRNA-ROR gene. The overexpression regulates epithelial–mesenchymal transition and leads to the activation of the Notch signaling cascade, which is directly linked to tumor metastasis [66].

According to Xiangyi Ma et al. 2024 [1], the upregulation of histone modifications at chr12p13.32—including methylation (H3K4me, H3K4me3) and acetylation (H3K9ac, H3K27ac)—activates the expression of lincRNA GAU1 and the oncogene *GALNT8*, both of which contribute to retinoblastoma progression [67].

#### 3.2.2. Histone Deacetylase (HDAC)-Dependent Oncogenic Effects

In contrast to the pro-oncogenic effects of excessive acetylation, histone deacetylases (HDACs) contribute to tumor progression through an opposing yet equally pathological mechanism: transcriptional repression of regulatory pathways. HDAC overexpression in retinoblastoma is linked to suppression of apoptotic mediators, disruption of DNA repair fidelity, and altered control of cell cycle regulators. According to Malwina Lisek et al. 2024 [31] deacetylases play a significant role in various stages of cancer and are associated with different types of solid and hematological cancers, including retinoblastoma. Deacetylases interact with many proteins involved in the cell cycle, such as cyclin-dependent kinases (CDKs), cyclins, p21, p27, pRb, A-kinase anchoring protein (or PKA) (AKAP95), as well as pro- and anti-apoptotic proteins (e.g., FAS receptor/APO1-FASL linker, tumor necrosis factor (TNF) receptors (TNF-TNF), TNF-related apoptosis-inducing ligand (TRAIL-TRAIL) receptors, cytoplasmic FLICE-like inhibitory protein (c-FLIP), DISC complex components (death-inducing signaling complex), caspase 8, Bcl-2 (B-cell lymphoma 2), Bax (Bcl-2-associated X protein), AIF (apoptosis-inducing factor), or Apaf-1 (apoptotic protease activating factor 1)), and thus exert effects on oncogenesis [68]. Additionally, deacetylases are involved in DNA damage repair mechanisms. Specifically, they deacetylate histones H3K56 and H4K16, thereby regulating processes such as non-homologous end joining, DNA excision repair, and double-strand break repair [69]. They also regulate the activity of proteins involved in these processes, such as ATR (ataxia-telangiectasia and Rad3-related), ATM (ataxia-telangiectasia mutated), BRCA1 (breast cancer susceptibility protein 1), FUS (fused in sarcoma), 53BP1 (p53-binding protein), MSH2 (MutS homolog 2), PARP-poly (ADP-ribose) polymerase, and Ku70 (lupus autoantigen protein Ku p70). Class I deacetylases interact with the Rb protein and suppress the transcription of E2F, which is vital for cell cycle regulation. This interaction causes epigenetic silence of transposable elements in the genome. Based on Malwina Lisek et al. 2024 [31] and Na Yu et al. 2019 [32], the oncogene c-Myc, a member of the *MYCN* family of genes that regulate several genes associated with proliferation, metastasis, and cell survival was underexpressed in the Wageningen–Erlangen (WERI)-RB1 and Y79 cases, even though it is typically overexpressed in various types of cancer. The administration of deacetylase inhibitors, particularly trichostatin A, resulted in increased expression of the gene only in the first case, where it caused morphological changes in the cells and reduced their viability. In the Y79 case, transcription of *c-Myc* was not increased. The administration of exogenous c-Myc also inhibits cell proliferation and reduces their viability. Deacetylase 2 mainly regulates the transcription of the gene, which was reduced with the administration of inhibitors, thus proving that it decreases the transcription of the gene in retinoblastoma cases. Finally, according to Malwina Lisek et al. 2024 [31], in most patients with retinoblastoma, increased expression of class 3 deacetylases, Silent Information Regulator 1 (SIRT1), SIRT2, and SIRT6, was observed. The expression of the first one is also influenced by the proto-oncogene MYCN [31].

The Jun Sun et al. 2019 [35] study found that WT161 is an inhibitor of histone deacetylase 6, which induces apoptosis of retinoblastoma cells. Specifically, this factor increases histone H3 and H4 acetylation on Bad promoters in cancerous cells. As a result, the Bad gene is overexpressed, and retinoblastoma cells die. So, this inhibitor can be used as a treatment factor for retinoblastoma, since it leads to apoptosis of retinoblastoma cells, and this approves the pathological effect of HDAC in oncogenesis.

The Yiting Zhang et al. 2016 [36] study also states that the levels of HDAC in cancer have increased. HDAC 9, which belongs to type II HDACs, conserve serine residues in the amino-terminal domain, which can be phosphorylated. In retinoblastoma cases, HDAC 9 is increased and treatments which can downregulate it could be effective [68]. Specifically, limited expression of these HDACs suppresses cell cycle progression and diminishes the ability to produce clonogens and tumors in experimental animals. So, this research, as well as the others, confirms the impact of HDACs in oncogenesis and the selection of these proteins as targets for therapeutic interventions.

The most important findings regarding histone acetylation are presented at Table 2.

**Figure 2:**
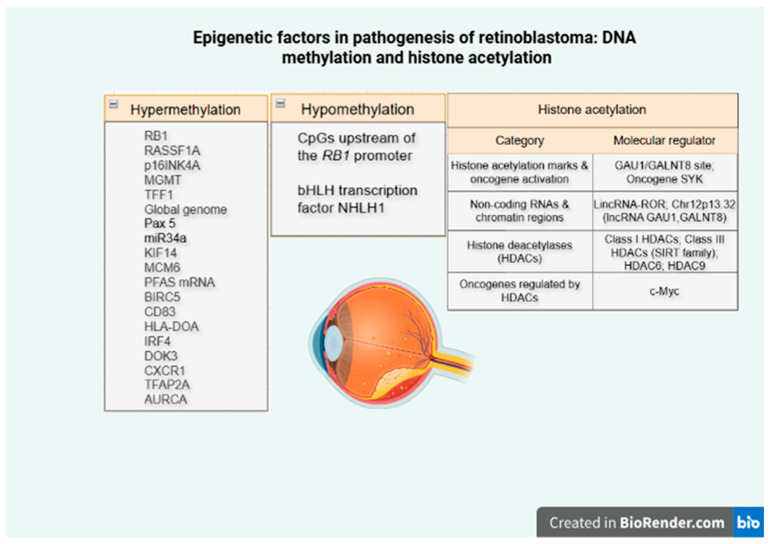
Epigenetic regulation in retinoblastoma: methylation and acetylation.

To visually summarize the interplay of epigenetic alterations in retinoblastoma, we provide a schematic illustrating how methylation and histone acetylation drive tumor initiation and progression (Figure 2).

## 4. Discussion

Epigenetic modifications such as DNA methylation and histone acetylation are key regulators of gene expression and chromatin structure, significantly impacting cancer development and progression, including retinoblastoma [70,71], demonstrating that comprehensive genomic and epigenetic profiling of retinoblastoma tumors can reveal candidate genes and pathways that are epigenetically silenced but potentially reversible. This study [70] identified that SYK, a non-receptor tyrosine kinase, is involved in signaling pathways that regulate cell proliferation, differentiation, and survival. In the context of retinoblastoma, aberrant acetylation at the *SYK* promoter enhances its expression, promoting oncogenic signaling and tumor cell survival. Also, F pharmacologic inhibition of *SYK* induces apoptosis in retinoblastoma cells, underscoring its potential as a therapeutic target [71].

Aberrant DNA methylation, often leading to the silencing of tumor suppressor genes, is a well-established mechanism in retinoblastoma tumorigenesis [72,73]. Recent studies have identified specific DNA methylation patterns that not only contribute to tumor initiation but also correlate with clinical outcomes, making them potential biomarkers for diagnosis and prognosis [26]. Aberrant DNA methylation has also been accepted as a feature across multiple cancer types [74,75]. Epigenetic silencing is more frequently observed in certain cancer types, often aligning with the distribution of genetic mutations, which suggests that both tumor suppressor gene inactivation and genetic alterations can contribute to cancer development. For example, except for retinoblastoma, *MutL Homolog 1 (MLH1)* is frequently silenced in colorectal and endometrial cancers [76,77,78], while methylation of *BRCA1* and *RAD51C* is commonly detected in ovarian and breast cancers [73]. Regarding *MLH1*, epigenetic loss of *MLH1* is observed in approximately 20% of endometrial cancers, correlating with larger tumor volume, lymph node positivity, and reduced recurrence-free survival in multiple cohorts [78,79]. Interestingly, in early-stage diseases, *MLH1* methylation is highly prevalent (~70%) but not uniformly predictive of recurrence, highlighting stage-dependent prognosis [80,81,82,83]. *Adenomatous Polyposis Coli (APC)* promoter methylation is a characteristic of gastrointestinal cancers [84], and hypermethylation of the CDKN2A/p16 promoter leads to its inactivation in esophageal adenocarcinoma.

In this context, recent studies have further validated *Transcription Factor Activating Enhancer-Binding Protein 2 Alpha (TFAP2A)* promoter methylation as a highly specific biomarker in aqueous humor (92.7% diagnostic accuracy) for distinguishing retinoblastoma from benign retinal conditions [29,85,86]. Moreover, genome-wide methylation profiling of aqueous humor cfDNA has defined robust subtypes of retinoblastoma linked to clinical outcomes, identifying differentially methylated genes (e.g., *TFF1*, *BCHE*, *PCDHB2*, *ADAM33*) that correlate with aggressive disease and chemoresistance. On a mechanistic level, elevated DNMT expression [46,87,88,89,90] and increased global DNA methylation in peripheral blood and tumor tissue suggest a co-oncogenic role of methylation in retinoblastoma beyond *RB1* mutations [26].

Genome-wide profiling further reveals dozens of hypermethylated tumor suppressors (e.g., *RB1*, *CDKN2A*, *RASSF1A, MLH1*, etc.), reinforcing the idea of epigenetic cooperativity in retinoblastoma pathogenesis [39]. In other tumor types, such as colorectal and endometrial cancers, *MLH1* promoter methylation serves as a frequent second-hit silencing event in sporadic and Lynch-associated disease [91]. Likewise, *BRCA1* methylation in ovarian cancer (~16%) mirrors *BRCA1* genetic loss and correlates (in certain assay types) with treatment response, supporting methylation as both a functional alteration and a biomarker across cancers [92].

In parallel with DNA methylation, histone acetylation—regulated by histone acetyltransferases (HATs), enzymes that acetylate histones, and histone deacetylases (HDACs), enzymes that remove acetyl groups from histones—plays a pivotal role in chromatin remodeling and regulation of gene expression. Disruptions in these epigenetic mechanisms have been increasingly associated with the development of various forms of cancer [31,69]. In retinoblastoma, studies have shown that dysregulation of HDAC activity disrupts the cell cycle, promoting uncontrolled cell proliferation and survival [65,66]. Pharmacological inhibition of HDACs exhibits anticancer activity [93,94]. Compounds such as vorinostat induce apoptosis and cell cycle arrest [89], while belinostat has demonstrated potent anticancer activity, even eliminating vitreous seeds without retinal toxicity in animal models [94]. Although several inhibitors are still in the preclinical or clinical stages of evaluation, their understanding is essential due to the complexity of their use and combination [95].

Bromodomain and Extra-Terminal Domain (BET) proteins (BRD2, BRD3, BRD4, BRDT) also act as epigenetic readers of acetylated lysines, regulating oncogene expression and cell proliferation, thus constituting attractive targets for anticancer therapy [96]. BET inhibitors, such as the drug JQ1, prevent an epigenetic reading by these proteins, leading to mitotic dysfunction, impaired meiosis, and inhibition of angiogenesis [97].

While not specific to retinoblastoma alone, HDACs and HATs exhibit genetic- and expression-level abnormalities in many types of cancer, including lung adenocarcinoma and hematological malignancies [98,99]. HDACs such as HDAC1, HDAC3, and HDAC6 are frequently overexpressed in solid tumors, promoting malignant phenotypes through the repression of tumor suppressor genes, enhancement of epithelial–mesenchymal transition (EMT), immune evasion, and increased angiogenesis [100,101]. Furthermore, abnormal acetylation of histone H3 has been associated with tumor progression and poor prognosis across various cancers [102].

Resistance to conventional therapies, particularly cisplatin, has been linked to disrupted acetylation patterns, suggesting that HDAC inhibitors may help overcome chemoresistance [103]. HDAC inhibitors such as entinostat, panobinostat, and belinostat, as well as novel isoform-specific compounds, show synergistic effects when combined with chemotherapy or immunotherapy [94,104,105]. Among them, entinostat (MS-275) selectively targets class I HDACs, restoring histone acetylation and reactivating tumor suppressor genes, while enhancing tumor immunogenicity [104]. Preclinical studies in retinoblastoma models have also shown encouraging results: the HDAC6 inhibitor WT161 induces apoptosis and sensitizes tumor cells to cisplatin [35], whereas intravitreal belinostat effectively eradicates vitreous seeds with minimal retinal toxicity. Although DNMT inhibitors have not yet been evaluated directly in retinoblastoma, their combination with HDAC inhibitors has demonstrated synergistic effects in other cancers [94]. Overall, epigenetic-based therapies hold promise as adjuncts to conventional treatments, potentially improving outcomes and overcoming resistance in retinoblastoma.

Moreover, recent research has explored combination therapies targeting both DNA methylation and histone deacetylation pathways. For example, combining HDAC inhibitors with DNA methyltransferase inhibitors (DNMTis) holds promise for reactivating silenced tumor suppressor genes more effectively, thereby suppressing tumor growth and metastasis. Particularly, inhibition of *UHRF1*, a key epigenetic regulator coordinating DNA methylation and histone modifications, sensitizes retinoblastoma cells to HDAC inhibitors, suggesting novel therapeutic avenues targeting this interplay [31].

Genome-wide methylation profiling via aqueous humor liquid biopsy revealed methylation patterns strongly associated with disease stage and prognosis, indicating its potential as a non-invasive prognostic biomarker [26]. Also, elevated global DNA methylation levels and increased expression of DNA methyltransferases (DNMT1, DNMT3A, DNMT3B) have also been linked with poor prognosis and higher tumor aggressiveness [39]. Furthermore, promoter methylation of genes such as *RASSF1A* (59%) and *MGMT* (15%) has been correlated with reduced survival and higher metastatic risk [3].

In summary, targeting epigenetic modifications via DNA methylation and histone acetylation inhibitors represents a promising strategy for improving retinoblastoma treatment. As ongoing research elucidates detailed mechanisms and optimizes therapeutic combinations, these epigenetic therapies hold potential for more personalized and effective interventions in pediatric oncology. Furthermore, epigenetics could provide significant information about the prognosis of retinoblastoma, but further research is necessary.

Our results should be interpreted with some caution given the limitations of our design. First, the articles included in this review were retrieved from MEDLINE and Scopus, so articles that are indexed exclusively in other databases are not represented herein. Secondly, this is a scoping review so the results were not analyzed quantitatively.

## 5. Conclusions

DNA methylation and histone acetylation are key epigenetic mechanisms linked to the pathogenesis of retinoblastoma. Dysregulation of these processes can lead to abnormal gene expression patterns that drive tumor initiation and progression. In particular, alterations affecting oncogenes and tumor suppressor genes play a central role in the development and behavior of cancer cells. In retinoblastoma, promoter hypermethylation of tumor suppressor genes can lead to their silencing. This results in uncontrolled cell proliferation and tumor growth. Similarly, excessive histone acetylation can modify chromatin structure and influence the transcriptional activity of critical genes. A better understanding of these epigenetic mechanisms is essential for improving diagnostic, therapeutic, and prognostic strategies. Since epigenetic changes are potentially reversible, they represent promising targets for drug development. Continued research in this area could contribute significantly to the advancement of personalized therapies and improved clinical outcomes for patients with retinoblastoma.

## Figures and Tables

**Table 1:** Synopsis of studies regarding DNA methylation in the pathogenesis of retinoblastoma.

#	Author Year	Population	Affected Structure	Observed Change	Result
[6,21]	Jie Sun et al., 2020 A M Raizis et al., 2021	[6]. review [21]. *n* = 25	CpG islands in the *RB1* gene	Hypermethylation of the promoter or first exon	Decreased expression of *RB1*
[23]	Özge Şükrüoğlu Erdoğan et al., 2024	*n* = 102 (50 retinoblastoma patients and 52 healthy individuals.)	*RB1* promoter	Not methylated	No association between methylation of *RB1* promoter and cancer
[21]	A M Raizis et al., 2021	*n* = 25	CpGs upstream of the *RB1* promoter	Hypomethylation	Decreased expression of *RB1*
[6]	Jie Sun et al., 2020	review	tumor suppressor genes *RASSF1A, p16INK4A, MGMT, TFF1*	Hypermethylation in genes or in their promoters	Decreased expression of the genes, Increased viability of retinoblastoma cells
[26]	Hülya Yazici et al., 2020	*n* = 113 (69 patients with retinoblastoma, 26 healthy siblings, 18 healthy unrelated children)	Global genome including tumor suppressor genes	Hypermethylated by DNMT1	Inactivation of tumor suppressor genes
[27]	Lan Jin et al., 2021	*n* = 20 patients with retinoblastoma	*Pax5* gene	Hypermethylated	Hyperexpression that inhibited proliferation and migration of retinoblastoma
[28]	Bo Yang et al., 2020	*n*= 62 Patients with retinoblastoma	miR34a	Hypermethylation due to CASC8	Decreased expression that inhibits cancer growth
[6,7]	Tatsiana Ryl et al., 2024, Jie Sun et al., 2020	[6]. review [7]. *n*= 59 samples from retinoblastoma patients	TFF1	Hypermethylated	Decreased expression, Increased viability of retinoblastoma cells
[18]	Yuyang Zeng et al., 2020	*n*= 127 (119 retinoblastoma samples, 8 healthy control samples)	*KIF14, MCM6* genes	Hypermethylation	Hypermethylated genes become oncogenes
[17]	Sipeng Zuo et al., 2023	not mentioned	N5-methylcytosine (m^5^C) PFAS mRNA	High global m^5^C levels Overexpression of PFAS modulated by NSUN2	Enhanced biosynthesis of purines
[58]	Peiyao Mao et al., 2022	*n* = 59 retinoblastoma samples	*BIRC5 (survinin)*, *CD83*, *HLA-DOA*, *IRF4*, *DOK3*, and *CXCR1* genes	Hypermethylated	Dysregulated expression compatible with tumorigenesis
[29]	Qi Zeng et al., 2021	not mentioned	CpG logi at *TFAP2A*, cfDNA	Hypermethylated	Dysregulated closure of neural tube
[61]	Hiroshi Tanaka et al., 2020	not mentioned	AURCA	Methylated	Degradation of p53
[7]	Tatsiana Ryl et al., 2024	*n* = 59 retinoblastoma samples	bHLH transcription factor NHLH1	Hypomethylated	Overexpression

# refers to the reference citation.

**Table 2:** Synopsis of studies regarding histone acetylation in the pathogenesis of retinoblastoma.

#	Author/Authors	Population	Affected Structure	Observed Change	Result
[2]	Linbin Zhou et al., 2024	Not mentioned	GAU1/GALNT8 site, Oncogene *SYK*	Acetylation of histones H3K9ac and H3K27ac, Acetylation of histone H3K9/14ac	Overexpression and Oncogenesis
[1,2]	Linbin Zhou et al., 2024 and Xiangyi Ma et al., 2024	[1]. review [2]. not mentioned	LincRNA-ROR	Acetylation of histone H3K27	Increased production and oncogenesis
[1]	Xiangyi Ma et al., 2024	review	Chr12p13.32	Overacetylation of histones H3K4me, HEK4m3, HEK9ac and HEK27ac	Overexpression of IncRNA GAU1 and oncogene *GALNT8*
[31]	Malwina Lisek et al., 2024	review	Cell cycle’s proteins	Interaction with HDACs	Oncogenesis
			DNA damage repair mechanism	Interaction with HDACs	Oncogenesis
			Rb protein	Interaction with class I HDACs Overexpression	Decreased transcription of *E2F*
			Class 3 Deacetylases		Retinoblastoma
[31,32]	Malwina Lisek et al., 2024 and Na Yu et al., 2019	[31]. review [32]. not mentioned	Oncogene c-Myc	Interaction with HDAC 2	Underexpression in two specific retinoblastoma cases
[35]	Jun Sun et al., 2019	Not mentioned	HDAC 6	Interaction with WT161	Retinoblastoma cell apoptosis
[36]	Yiting Zhang et al., 2016	*n* = 55 (50 retinoblastoma samples, 5 healthy controls)	HDAC 9	Increased expression	Retinoblastoma

# refers to the reference citation.

## Data Availability

The original contributions presented in this study are included in the article. Further inquiries can be directed to the corresponding authors.
